# Supplementary material for: The alternative oxidase reconfigures the larval mitochondrial electron transport system to accelerate growth and development in Drosophila melanogaster
Source: bioRxiv. 2025 Feb 21:2025.02.20.639223. Preprint. [Version 1] doi: 10.1101/2025.02.20.639223 (PMC11870600; doi:10.1101/2025.02.20.639223)
Supplement: 1 [file NIHPP2025.02.20.639223V1-supplement-1.pdf]

Supplemental Material

The alternative oxidase reconfigures the larval mitochondrial electron transport system to accelerate growth and development in *Drosophila melanogaster*

Geovana S. Garcia, Murilo F. Othonicar, Antonio Thiago P. Campos, Eric A. Kilbourn, Kênia C. Bícego, Johannes Lerchner, Jason M. Tennessen & Marcos T. Oliveira

Supplemental Table

Table S1: Larval agitation and prepupal heat burst events are increased by AOX. The average noise of the voltage change output of the chip calorimeter (σ, see representative output in Figure 1C) +/- standard error represents larval movements inside the calorimeter chambers. The data was obtained from two biological replicates, using 6-10 individuals in each one (see details in Material and Methods). The average number of prepupal heat bursts per cycle and the percentage of cycles with heat bursts per individual were calculated for 10 individuals from each genotype in the ~18 min cycle of each real-time measurement of the calorimetry assays.

	Control	AOX
Larval σ	0.5 ± 0.04 μV	0.9 ± 0.1 μV*
Prepupal heat bursts/cycle	0.467 ± 0.142	1.47 ± 0.213[£]
% cycles with heat bursts/individual	33.3 ± 38.5	73.3 ± 30.6*

* significantly different ($p < 0.05$) according to a Student's t-test.

[£] significantly different ($p < 0.001$) according to a Mann-Whitney U test.

Supplemental Figure Legends

Figure S1. Effects of AOX expression on development. (A) Relative transcript levels of *AOX*, *Gpo1* and *Ldh* in wandering L3 larvae. Transcript levels were estimated using the levels of the housekeeping *eIF-1A* transcript as reference, and were normalized (arbitrarily set to 1.0) for *AOX* by its levels in AOX-expressing larvae (*3xtubAOX*) at 25 °C, and for *Gpo1* and *Ldh* by their levels in control larvae (w^{1118}) also at 25 °C. The data represent the average of two independent biological replicates, and the error bars standard deviations. Egg-to-pupa developmental time (B) and larval body masses (C) at the indicated temperatures for the background control w^{1118} line and the red-eye control *UAS-empty*^{2nd}*;UAS-empty*^{3rd} line, showing no effects of the *white* gene. (D) Representative results of in-gel activity for complex II (see Figure 3 for data quantitation). Larval body masses (E) and egg-to-pupa developmental time (F,G) of the w^{1118} and *3XtubAOX* lines cultured on dietary supplementation with the uncoupler CCCP (E,F) and the CI inhibitor rotenone (G) at the indicated temperatures. Data represents the average of two-three biological replicates, each with at least five-ten technical replicates. * indicates significant differences according to Student's t-test ($p < 0.05$). Two-way ANOVAs, followed by Tukey's *post hoc* tests, were used to show differences ($p < 0.05$) between genotypes, between temperatures (denoted by the symbols < or =), or in the interaction genotype X temperature (denoted by the letters 'a-c').

Figure S2. AOX significantly contributes to larval respiration, particularly to the Leak state. Remaining oxygen consumption rates (OCR) driven by the indicated dehydrogenase at the indicated temperature after inhibition of AOX with propyl gallate (A, C and E), or of CIII with antimycin A (B, D and F). The data represent percentages of the

total OCR as shown in Figure 3A-C. The mitochondrial respiratory states OXPHOS (A and B), ET-capacity (C and D) and Leak (E and F) are shown. * indicates a significant difference ($p < 0.05$) between AOX and control larval samples, according to Student's t-tests.

Figure S3. Metabolic analyses of AOX-expressing lines. (A) Distribution of τ_m of free NADH autofluorescence (left graph) and of reverse amplitude rate of enzyme-bound/free NADH (a2/a1, right graph) of samples of larval fat bodies of each genotype. (B) Partial Least Squares Discriminant Analysis (PLS-DA) plots showing separation among the metabolic profiles of mid-L2 and wandering L3 larvae of the indicated genotypes, based on liquid chromatography-mass spectrometry. (C,D) Tendency of AOX dose-dependent increase in NAD+ and ATP levels, and in the NAD+/NADH and ATP/ADP ratios. (E) Heatmaps indicating the relative levels of the identified glycolytic and tricarboxylic acid cycle metabolites.

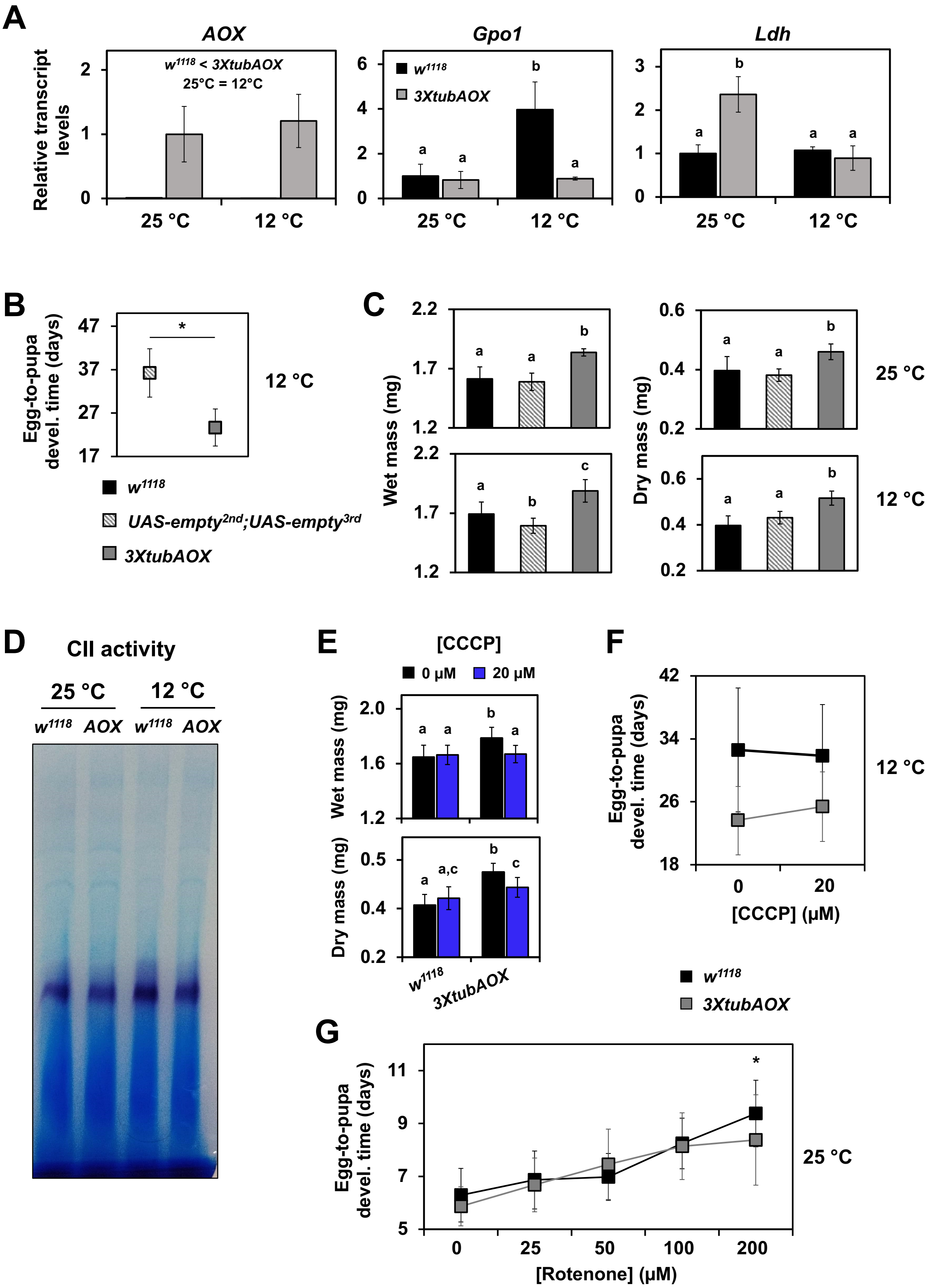

Supplemental Figure S1.

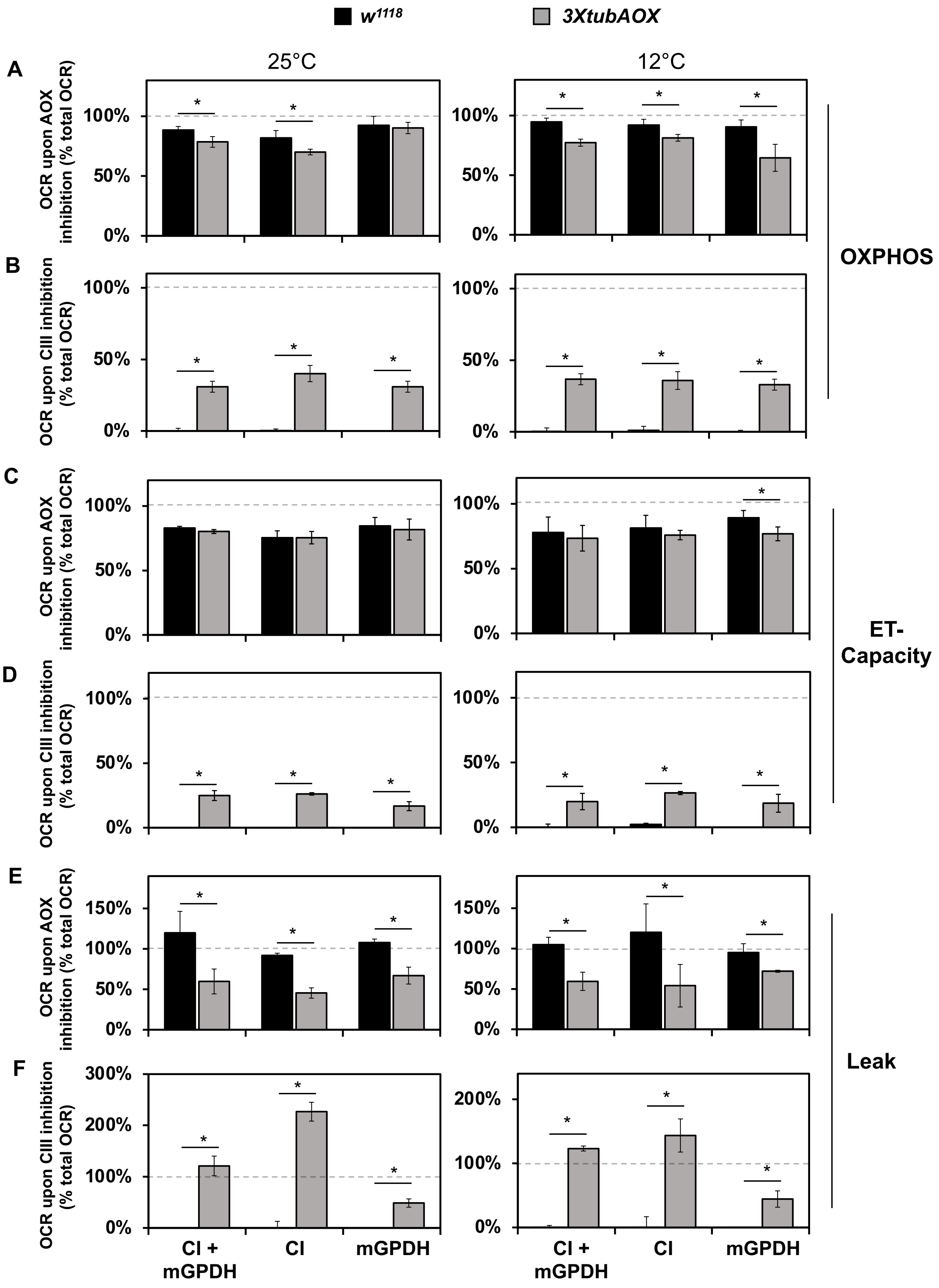

Supplemental Figure S2.

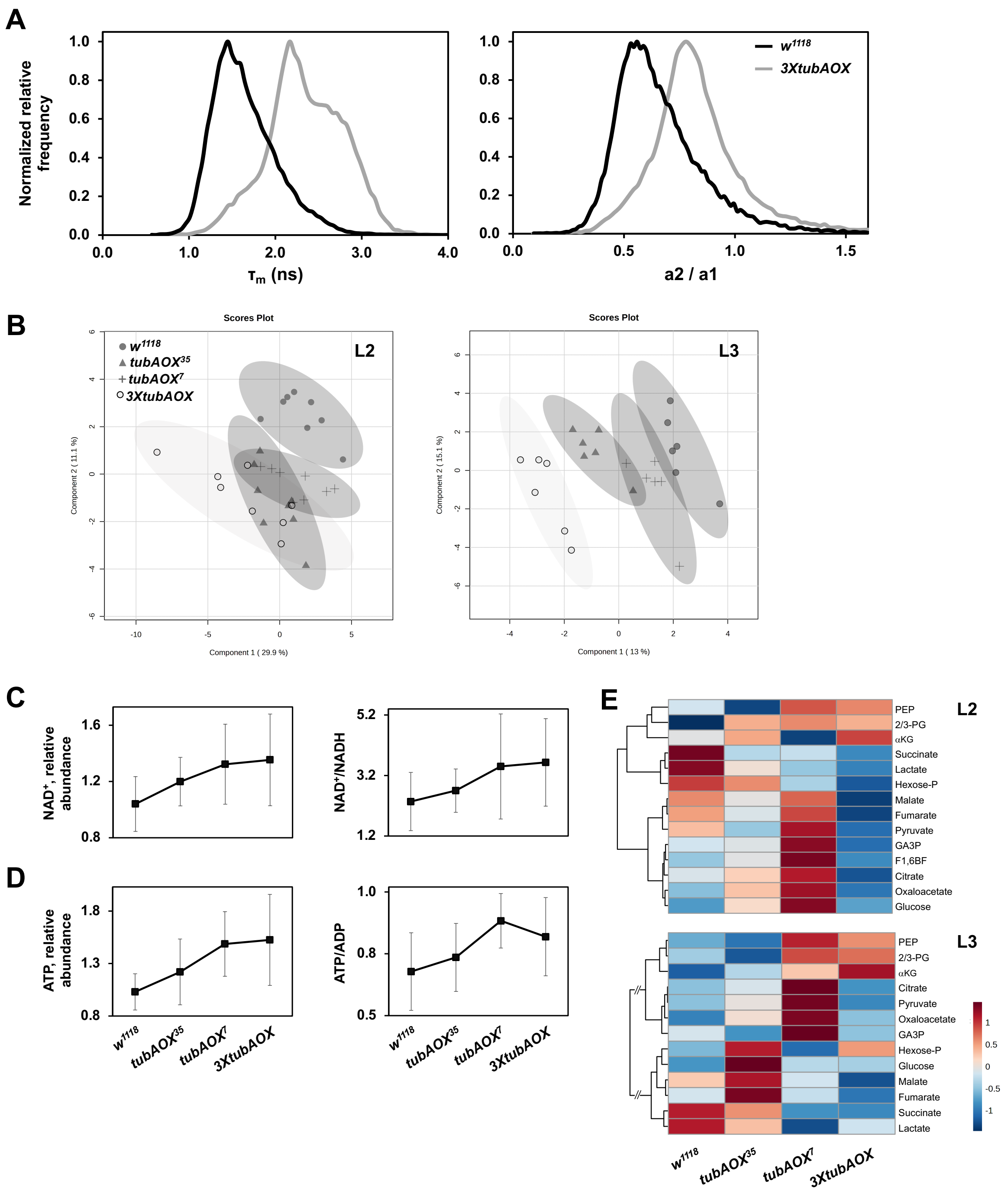

Supplemental Figure S3.
